# Loneliness and socio-demographic predictors of life satisfaction among Saudi young adults

**DOI:** 10.3389/fpsyg.2026.1828590

**Published:** 2026-05-25

**Authors:** Mohammad Saud Alotaibi

**Affiliations:** Department of Social Work, College of Social Sciences, Umm Al-Qura University, Mecca, Saudi Arabia

**Keywords:** life satisfaction, loneliness, psychological wellbeing, Saudi Arabia, subjective wellbeing, young adults

## Abstract

Loneliness has emerged as a growing public health concern with profound implications for subjective wellbeing, yet its relationship with life satisfaction remains insufficiently examined among young adults in Arab societies, particularly in Saudi Arabia. This study investigated the association between loneliness and life satisfaction, and explored the socio-demographic predictors of life satisfaction among Saudi young adults. A cross-sectional survey design was employed with a convenience sample of 646 participants aged 18–25 years recruited from Saudi Arabia. Loneliness was measured using the UCLA Loneliness Scale (ULS-8), and life satisfaction was assessed using the Satisfaction with Life Scale (SWLS). Spearman correlation, Kruskal–Wallis tests, and a Generalized Linear Model (GLM) were used to examine associations and identify significant predictors. Results revealed a significant negative association between loneliness and life satisfaction. GLM analysis identified loneliness and income level as the strongest predictors of life satisfaction, while gender and age group were not significant predictors. These findings establish an empirical foundation for understanding the contexts in which loneliness most acutely undermines wellbeing among Saudi young adults, with implications for social work practice and the design of community-based interventions aimed at fostering belonging and social integration.

## 1 Introduction

Life satisfaction is defined as a global cognitive evaluation whereby individuals assess the overall quality of their lives according to their own standards, goals, and expectations (Diener et al., 1985; Pavot and Diener, 2008). As the cognitive component of subjective wellbeing, it reflects a global and relatively stable evaluative judgment about one's life as a whole, distinct from temporary emotional experiences, and conceptually paired with positive and negative affect as its affective counterparts (Busseri and Sadava, 2011; Diener, 1984; Diener et al., 1985). Diener et al. (1985) proposed one of the most influential theoretical approaches in this field, the tripartite model of subjective wellbeing, which distinguishes between life satisfaction as a cognitive appraisal and positive and negative affect as emotional components. This model has guided a substantial body of research and continues to inform how life satisfaction is conceptualized and measured across different populations (Pavot and Diener, 2008).

Previous research has identified several factors that influence life satisfaction, including demographic characteristics such as gender (Joshanloo and Jovanović, 2020), age (Baird et al., 2010; Morgan and O'Connor, 2017), and income (Jebb et al., 2018). With respect to gender, findings across studies are not always consistent. A large-scale cross-national study involving over 1.8 million participants from 166 countries found that although women tended to report greater life satisfaction than men across most demographic groups, this pattern varied by age and region, suggesting that gender differences in life satisfaction are context-dependent rather than universal (Joshanloo and Jovanović, 2020). Age has also been linked to differences in life satisfaction, though the nature of this relationship is not consistent across studies. Some research reports relative stability across adulthood, while others identify declines in very old age or non-linear patterns depending on the population and methods used (Baird et al., 2010; Morgan and O'Connor, 2017). Notably, evidence drawn from approximately 299,000 respondents across 145 countries indicates that the period between ages 15 and 24 represents the most rapid decline in life satisfaction across the lifespan, with the notable exception of South Asia and the Middle East and North Africa (MENA), where this pattern of decline was found to be less pronounced (Handa et al., 2023). Regarding income, a large-scale study drawing on data from more than 1.7 million respondents across 164 countries found that life satisfaction peaks at a certain income level and does not continue to rise with further increases in wealth, suggesting diminishing returns beyond a point of material sufficiency (Jebb et al., 2018).

Loneliness is “the unpleasant experience that occurs when a person's network of social relations is deficient in some important way, either quantitatively or qualitatively” (Perlman and Peplau, 1981, p. 35). This deficiency may take the form of social loneliness, reflecting an absence of a broader social network, or emotional loneliness, reflecting the lack of close and intimate relationships (Weiss, 1973). Among young people, loneliness has been identified as a growing concern; a cross-temporal meta-analysis of 345 studies found that loneliness levels among emerging adults increased significantly over a 43-year observation period spanning from 1976 to 2019 (Buecker et al., 2021). Its relationship with life satisfaction is consistently negative across different cultural contexts, including Turkey (Ozben, 2013), Indonesia (Raichani Tohitin and Qudsyi, 2025), and the Middle East (Al Omari et al., 2023). Within the Saudi context specifically, this association has been documented among female university students aged 18 to 25, where higher loneliness was negatively associated with life satisfaction (Alqahtani et al., 2020).

The association between loneliness and life satisfaction is not uniform across cultures. In individualistic societies, autonomy and personal choice in social relationships have greater implications for wellbeing, such that social disconnection carries less cultural weight (Lykes and Kemmelmeier, 2014). In collectivistic societies, by contrast, traditional social bonds and family cohesion are fundamental to wellbeing, such that their absence is more strongly linked to loneliness (Lykes and Kemmelmeier, 2014; Taniguchi and Kaufman, 2022), and the association between loneliness and life satisfaction is more pronounced than in individualistic contexts (Goodwin et al., 2001).

Despite growing interest in wellbeing research within Saudi Arabia, studies examining the combined roles of gender, age, income status, and loneliness as predictors of life satisfaction among young adults remain scarce. This gap is particularly noteworthy given that Saudi Arabia has undergone significant social, economic, and cultural transformation over the past two decades (Burger and Arampatzi, 2025), changes that may meaningfully shape how young adults in this context experience and evaluate their lives. While some research has begun to examine the relationship between loneliness and life satisfaction among Saudi young adults, existing studies have been limited to female samples (Alqahtani et al., 2020), leaving a gap in understanding how this relationship operates across both genders. This is especially significant given that young adulthood is a developmentally critical period characterized by concurrent academic, occupational, and social transitions, all of which may heighten vulnerability to loneliness and shape how individuals appraise their overall life quality.

The present study therefore aims to examine the predictive roles of gender, age, self-reported income status, and loneliness in explaining life satisfaction among Saudi young adults aged 18 to 25 years. Specifically, it seeks to determine the extent to which these variables significantly predict life satisfaction, with particular attention to the role of loneliness as a psychological predictor alongside socio-demographic factors, given the collectivist cultural context of Saudi Arabia in which social belonging constitutes a core determinant of wellbeing. Based on the reviewed literature, the following hypotheses were formulated:

H1: Loneliness is expected to be significantly and negatively associated with life satisfaction among Saudi young adults.

H2: Life satisfaction is expected to differ significantly based on socio-demographic and psychological categorizations among Saudi young adults.

H3: Loneliness is expected to be significantly and independently associated with life satisfaction when controlling for socio-demographic variables among Saudi young adults.

## 2 Methods

### 2.1 Participants and sampling

This study employed a cross-sectional survey design. Participants were recruited using a convenience sampling method and were eligible to take part if they were Saudi nationals between 18 and 25 years old, and agreed to participate voluntarily. The required minimum sample size was set at 384 participants using Cochran, 1977 formula, based on a 95% confidence level and a 5% margin of error, which was considered sufficient for achieving appropriate statistical power. In addition, an a priori power analysis conducted through G^*^Power 3.1 (Faul et al., 2007) supported this estimate, indicating that the sample size could detect medium effect sizes (*f* = 0.25) at α = 0.05 with 95% power. In total, 652 individuals were recruited, but six were excluded due to incomplete questionnaire data, leaving 646 participants for the final analysis. The final sample size exceeded the minimum requirement, supporting the adequacy of the study's statistical power.

### 2.2 Measures

Demographic information was collected through a structured questionnaire that included gender, age, and income status. Income status was assessed based on participants' self-reported perception of their financial situation, categorized as low, moderate, or high.

#### 2.2.1 Satisfaction With Life Scale

The Satisfaction With Life Scale (SWLS) was used to assess participants' overall cognitive evaluation of life satisfaction. Originally developed by Diener et al. (1985), as a five-item, seven-point Likert scale designed to capture global judgments of satisfaction with one's life, the SWLS is a widely used self-report measure with sample statements such as “In most ways my life is close to my ideal” and “I am satisfied with my life.” The Arabic version of the scale, translated and validated within Saudi samples by Al-Dossary (2022), was adopted in the present study. Although the original scale uses a seven-point response format, a five-point Likert scale ranging from 1 (strongly disagree) to 5 (strongly agree) was employed to enhance response clarity in the online survey context, as psychometric evidence demonstrates adequate factor structure, reliability, and convergent validity when implemented with five response options (Espejo et al., 2022). In the current study, the scale yielded high internal consistency (Cronbach's α = 0.85), further supporting its suitability for use with this population.

#### 2.2.2 University of California, Los Angeles Loneliness Scale (UCLA-LS, 8-item version, ULS-8)

The 8-item short-form UCLA Loneliness Scale (ULS-8) was employed to assess participants' perceived levels of loneliness. This abbreviated measure is derived from the revised version of the UCLA Loneliness Scale (Version 3) developed by Russell (1996) and includes eight statements reflecting the frequency of loneliness-related experiences. Sample items include “I lack companionship” and “There is no one I can turn to.” Participants rated each statement using a 4-point Likert scale, ranging from 1 (never) to 4 (often). To ensure consistent scoring, positively worded items (e.g., “I am an outgoing person” and “I can find companionship when I want it”) were reverse-coded, so that higher overall scores indicated greater loneliness. The study used the Arabic version of the ULS-8 adopted from Alateeq et al. (2022), which has demonstrated acceptable reliability and validity in the Saudi Arabian population. In the present sample, the scale showed satisfactory internal consistency (Cronbach's α = 0.79), supporting its appropriateness for the current research.

### 2.3 Data collection procedure

Data were gathered through an online, self-administered questionnaire designed and delivered in Arabic using Microsoft Forms. The questionnaire link was disseminated via social media platforms to maximize accessibility and reach. All participants were provided with information regarding the study objectives, the voluntary nature of participation, the anonymity of responses, and the intended academic use of the data. Electronic informed consent was obtained prior to proceeding with the survey. Participants were informed of their right to withdraw at any stage without penalty, and complete confidentiality was maintained as no personally identifiable information was collected. Completing the questionnaire required approximately 15 min.

### 2.4 Data analysis

Statistical analyses were performed using IBM SPSS Statistics (Version 21) following a structured, multi-step procedure. As an initial step, the dataset was examined for extreme values. All scores obtained on the UCLA Loneliness Scale and the Satisfaction With Life Scale (SWLS) fell within the acceptable standardized range of |*Z*| ≤ 3.29, in accordance with the guidelines proposed by Tabachnick and Fidell (2007). Normality assumptions were subsequently assessed using the Kolmogorov–Smirnov and Shapiro–Wilk tests. Because both measures showed significant departures from normality (*p* < 0.05), non-parametric statistical techniques were employed throughout the analyses.

Descriptive indicators, including frequencies, percentages, and mean ranks, were computed to summarize the socio-demographic characteristics and psychological variables of the participants. The relationship between perceived loneliness and life satisfaction was examined using Spearman's rho (ρ) correlation analysis. In addition, a Generalized Linear Model (GLM) was estimated to determine whether loneliness significantly predicted life satisfaction. The model was specified with a Normal probability distribution and an Identity link function, which allows for direct interpretation of the B coefficients in the original scale of the SWLS. Robust covariance matrix estimation (COVB=ROBUST) was applied to correct for the violation of normality and ensure valid standard errors and *p*-values.

Group comparisons in life satisfaction across socio-demographic categories and levels of loneliness were conducted using non-parametric tests. The Mann–Whitney U test was applied to dichotomous variables (e.g., gender), whereas the Kruskal–Wallis H test was used for variables comprising three or more groups (e.g., income level and categorized loneliness scores). When the Kruskal–Wallis test yielded statistically significant results, Dunn–Bonferroni adjusted pairwise comparisons were carried out to identify specific group differences while controlling for inflation of Type I error.

To complement statistical significance testing, effect sizes were calculated for all significant findings. For Mann–Whitney analyses, the *r* statistic was reported, with interpretation benchmarks of 0.1 (small), 0.3 (medium), and 0.5 (large), as suggested by Tomczak and Tomczak (2014). For Kruskal–Wallis tests, eta squared (η^2^) was estimated using the formula η^2^ = χ^2^/(N−1). Interpretation followed Cohen's (1988) conventional benchmarks of 0.01 (small), 0.06 (medium), and 0.14 (large), providing an indication of practical significance beyond *p*-values.

## 3 Results

### 3.1 Correlational analysis

To examine the association between the primary study variables, a Spearman's rank-order correlation was conducted. As shown in Table 1, the analysis revealed a statistically significant, moderate negative relationship between perceived loneliness and life satisfaction (*r?* = −0.391, *p* < 0.01). This finding indicates that higher levels of loneliness are associated with lower levels of perceived life satisfaction among participants.

**Table 1:** Spearman's rho correlations UCLA loneliness and satisfaction with life.

Variable	UCLA loneliness scale
Satisfaction With Life Scale (SWLS)	−0.391^**^

** Correlation is significant at the 0.01 level (2-tailed).

### 3.2 Differential analysis of life satisfaction

Following the correlational findings, non-parametric tests were conducted to assess differences in life satisfaction across socio-demographic and psychological categories (see Table 2). The results demonstrated no statistically significant differences in life satisfaction based on gender (*p* = 0.191) or age (*p* = 0.750). In contrast, income status was significantly associated with life satisfaction, χ^2^(2) = 37.97, *p* < 0.001, η^2^ = 0.059, reflecting a moderate magnitude of association. *Post-hoc* comparisons using the Dunn–Bonferroni procedure revealed a clear hierarchical pattern (High > Medium > Low), where by participants in the high-income category reported significantly higher satisfaction compared to those in the middle- and low-income groups. Loneliness levels exhibited the strongest association with life satisfaction, χ^2^(3) = 84.84, *p* < 0.001, η^2^ = 0.131, indicating a large effect size. Participants reporting low levels of loneliness demonstrated substantially higher satisfaction ranks (Mean Rank = 416.03) compared to all other groups.

**Table 2:** Descriptive statistics and differential analysis of life satisfaction based on participants' characteristics and loneliness levels.

Variable	*n* (%)	Mean rank	Test statistic	*p*-value	Effect size	*Post-hoc*
Gender			U = 44877.50	0.191	–	–
Male	(35.6) 230	310.63				
Female	(64.4) 416	330.61				
Age			x^2^(3) = 1.21	0.75	–	–
19–18	(30.5) 197	321.06				
21–20	(47.2)305	320.11				
23–22	(12.8) 83	344.46				
25–24	(9.4) 61	319.84				
Income status			x^2^ (2)= 37.97	**<0.001**	*η^2^* = 0.059	High > Med > Low^*^
Low	(5.0) 32	157.69				
Medium	(83.7) 541	322.89				
High	(11.3) 73	400.72				
Loneliness levels			x^2^(3)= 84.84	**<0.001**	*η^2^* = 0.131	Low < All others^*^
Low levels	(28.9) 187	416.03				
Normal/moderate	(41.0) 265	312.9				
Moderate to high	(22.3) 144	268.28				
High levels	(7.7) 50	192.61				

*N* = 646.

U, Mann–Whitney U statistic; χ^2^, Kruskal–Wallis H statistic.

Effect sizes are reported as *r* (for Mann–Whitney tests) and η^2^ (for Kruskal–Wallis tests).

*Post-hoc* comparisons were conducted using the Dunn–Bonferroni correction; only statistically significant differences (*p* < 0.05) are presented.

* Significant pairwise differences confirmed across all group comparisons.

Bold values indicate statistically significant results (*p* < 0.05).

### 3.3 Predictive model for life satisfaction

To determine the unique contribution of each variable while controlling for others, a Generalized Linear Model (GLM) was estimated (Table 3). The omnibus test indicated that the model significantly improved model fit relative to the intercept-only model, χ^2^(7) = 133.65, *p* < 0.001. Loneliness emerged as the most influential predictor (Wald χ^2^ = 95.986, *p* < 0.001), with a negative regression coefficient (*B* = −0.309), indicating that each one-unit increase in loneliness is associated with a 0.309 unit decrease in life satisfaction, holding other variables constant. Income status also remained a statistically significant predictor. Relative to the high-income reference group, individuals in the low-income (*B* = −4.735, *p* < 0.001) and medium-income categories (*B* = −1.591, *p* = 0.008) differed significantly in their life satisfaction scores. Consistent with the non-parametric findings, gender and age did not make statistically significant contributions to the model.

**Table 3:** Generalized linear model (GLM) predicting satisfaction with life scale (SWLS).

Predictor	*B*	SE	95% wald CI	Wald χ2	*p*
(Intercept)	23.091	0.963	[21.204, 24.977]	575.384	**<0.001**
Gender (female)^a^	0.112	0.366	[−0.606, 0.830]	0.094	0.760
Age (18–19)^b^	−0.299	0.659	[−1.591, 0.993]	0.206	0.650
Age (20–21)^b^	−0.255	0.626	[-0.971, 1.481]	0.166	0.683
Age (22–23)^b^	0.167	0.719	[-1.577, 1.243]	0.054	0.816
Low income^c^	−4.735	0.885	[−6.469, −3.002]	28.662	**<0.001**
Medium income^c^	−1.591	0.596	[−2.758, −0.424]	7.138	**0.008**
Loneliness (UCLA sum)	−0.309	0.032	[−0.370, −0.247]	95.986	**<0.001**

*N* = 646; Dependent variable: SWLS total score.

Reference Groups: aMale, bAge 24–25, cHigh Income; coefficients indicate differences relative to these baseline groups.

Model specifications: Generalized linear model (GLM) with normal probability distribution and identity link function. robust covariance matrix estimation (COVB=ROBUST) was applied to ensure valid standard errors and *p*-values given the non-normal distribution of residuals.

Omnibus test: χ^2^(7) = 133.65, *p* < 0.001.

## 4 Discussion

The present study examined the predictive roles of gender, age, self-reported income status, and loneliness in explaining life satisfaction among Saudi young adults aged 18 to 25 years. The findings revealed that loneliness and income status were significant predictors of life satisfaction, whereas gender and age did not emerge as significant predictors. Notably, loneliness was the strongest predictor, demonstrating both a significant negative correlation and the largest effect size in the differential analysis, underscoring its central role in shaping life satisfaction among this population.

### 4.1 Loneliness as a psychological predictor of life satisfaction

The present study hypothesized a significant negative relationship between loneliness and life satisfaction among Saudi young adults. The findings supported this hypothesis, revealing a statistically significant negative association between loneliness and life satisfaction (*r*_*s*_ = −0.391, *p* < 0.01). This association was further reflected in meaningful differences in life satisfaction across loneliness levels, with participants reporting low loneliness demonstrating substantially higher life satisfaction compared to all other groups, yielding a large effect size (η^2^ = 0.131, Cohen, 1988). Consistent with these findings, loneliness also emerged as the strongest predictor of life satisfaction in the predictive model (*B* = −0.309, *p* < 0.001), indicating that each one-unit increase in loneliness was associated with a 0.309-unit decrease in life satisfaction when controlling for all other variables. These findings are consistent with evidence from diverse cultural contexts, including Turkey (Ozben, 2013), Indonesia (Raichani Tohitin and Qudsyi, 2025), Saudi Arabia (Alqahtani et al., 2020), and broader Middle Eastern samples (Al Omari et al., 2023).

Theoretically, this finding may be understood in light of Weiss (1973) conceptualization of loneliness as a deficiency in meaningful social and emotional bonds. Building on this, and interpreted through both the Belongingness Hypothesis (Baumeister and Leary, 1995; Mellor et al., 2008) and the Stress-Buffering Model (Cohen and Wills, 1985; Szkody et al., 2021), social relationships function not only as a core psychological need but also as a crucial buffer against stress. Consequently, their absence may foster loneliness and heightened emotional strain, which can, in turn, substantially undermine wellbeing and life satisfaction.

Beyond this, individuals who feel socially disconnected may also report lower self-esteem (Wong and Hamza, 2026) and reduced subjective wellbeing over time (Seifert, 2024), which can negatively affect how they evaluate their lives. This relationship may be especially strong in collectivist cultures such as Saudi Arabia, where social harmony and group cohesion are culturally emphasized (Hofstede, 2001). The present findings are consistent with cross-cultural evidence suggesting that the absence of close social and family bonds carries greater psychological cost in collectivistic societies than in individualistic ones (Lykes and Kemmelmeier, 2014; Taniguchi and Kaufman, 2022). In Saudi Arabia specifically, strong social and emotional bonds serve as a protective factor against loneliness, such that weakened social ties increase vulnerability to loneliness in this context (Alateeq et al., 2022). The observed association between loneliness and life satisfaction thus reflects not only a psychological dynamic but a culturally embedded one, in which social disconnection conflicts with deeply held expectations of communal belonging. Furthermore, longitudinal evidence from a six-wave study of Chinese university students found that loneliness negatively predicted subsequent life satisfaction at the within-person level (Chen et al., 2026), suggesting that the detrimental effect of loneliness on life satisfaction may persist over time. These longitudinal findings strengthen the interpretation of loneliness as a consistent negative correlate of life satisfaction. It should be noted, however, that the cross-sectional design of the present study precludes causal inference between loneliness and life satisfaction.

### 4.2 Socio-demographic factors and life satisfaction

The present study further tested whether income, gender, and age would significantly predict life satisfaction among Saudi young adults. With respect to income status, the findings revealed a statistically significant association with life satisfaction, characterized by a moderate effect size (η^2^ = 0.059, Cohen, 1988). A clear hierarchical pattern surfaced, where high-income participants reported significantly greater satisfaction than those in the medium and low-income categories. This was corroborated by the predictive model, in which (*B* = −4.735, *p* < 0.001) and medium-income (*B* = −1.591, *p* = 0.008) groups reported lower scores relative to the high-income reference.

These findings also revealed that financial status was a significant predictor of life satisfaction, with higher financial status associated with greater life satisfaction. This is consistent with prior research demonstrating that higher income levels are generally linked to more favorable life evaluations (Howell and Howell, 2008; Kahneman and Deaton, 2010), and that socioeconomic conditions predict life satisfaction among emerging adults specifically (Temizkan et al., 2023). In the Saudi context, this pattern may reflect the financial pressures experienced by young adults during the transition from education to the labor market, a period further shaped by the broader economic and social transformations underway in the Kingdom (Burger and Arampatzi, 2025). This interpretation may also be understood through the lens of aspiration-gap theory, which suggests that life satisfaction is shaped not only by objective income but by the perceived gap between individuals' income aspirations and their actual resources (Marton et al., 2026).

Contrary to expectations, however, neither gender nor age emerged as a statistically significant predictor, either in the differential analysis or in the predictive model. With respect to gender, this finding aligns with evidence that gender effects on life satisfaction are typically null or inconsistent, and tend to be shaped by cultural and contextual factors rather than gender alone (Fors Connolly, 2025; Joshanloo and Jovanović, 2020), a pattern similarly observed among young adults in Saudi Arabia (Abd Ellatif Elsayed and Aleriani, 2024), and may further reflect an ongoing convergence in life experiences between male and female young adults amid the social transformations associated with Vision 2030 (Burger and Arampatzi, 2025).

Regarding age, the absence of significant differences within the 18–25 range may suggest that this developmental period is characterized by broadly similar life circumstances, rendering age a less discriminating factor within this relatively narrow band of emerging adulthood (Baird et al., 2010). Notably, cross-national evidence suggests that the pattern of rapid life satisfaction decline observed among youth globally is less pronounced in the MENA region and South Asia (Handa et al., 2023), which may help explain the absence of significant age-related variation in the present sample.

### 4.3 Strengths and limitations

Several methodological strengths of the present study enhance confidence in the findings. The sample size (*N* = 646) substantially exceeded the minimum threshold established through both Cochran's formula and a priori power analysis, supporting the adequacy of statistical power. The use of validated Arabic versions of the SWLS and ULS-8, both of which have demonstrated acceptable psychometric properties within Saudi samples, strengthens the cultural appropriateness of the measures employed. The analytical approach combined multiple complementary statistical techniques, including Spearman correlation, non-parametric group comparisons with *post-hoc* testing, and a Generalized Linear Model, allowing for a comprehensive examination of both associative and predictive relationships. Effect sizes were systematically reported for all significant findings, providing an indication of practical significance beyond *p*-values. Furthermore, the inclusion of both male and female participants addresses a gap in the existing Saudi literature, which has predominantly relied on female-only samples (Alqahtani et al., 2020).

Despite the significance of the findings, several limitations should be noted. The cross-sectional design prevents causal inferences and does not clarify the temporal direction of the relationship between loneliness and life satisfaction. Data were collected using self-report measures, which may be subject to response biases or inaccuracies. Additionally, both key variables were self-reported and measured at the same time point, which may introduce common method variance. As a procedural measure, the two scales employed different response formats, specifically a four-point frequency scale for the ULS-8 and a five-point agreement scale for the SWLS, which may partially mitigate this concern. Future studies could address this limitation more rigorously through time-lagged or multi-source designs. Furthermore, the sample was limited to young adults aged 18–25 via convenience sampling, which limits both the representativeness of the findings and their generalizability to other age groups and broader populations. Finally, social media recruitment may exclude individuals who are not active on such platforms, and voluntary participation may favor those more willing to reflect on personal wellbeing.

### 4.4 Conclusion and recommendations

In conclusion, the present study examined the relationship between loneliness and life satisfaction among Saudi young adults, alongside the predictive roles of income, gender, and age. Loneliness emerged as the strongest independent predictor of life satisfaction, highlighting its particular significance in a collectivist cultural context where social bonds are central to meaning and fulfillment. Income was also a significant predictor, with lower-income participants reporting lower life satisfaction, reflecting the impact of economic vulnerability during this critical life stage. Neither gender nor age significantly predicted life satisfaction, suggesting that within this narrow developmental stage, these variables may be less discriminating factors.

Although the cross-sectional design limits causal inferences, the predictive model suggests a negative association in which loneliness is independently linked to lower life satisfaction, a pattern that warrants longitudinal investigation to confirm causal direction. These results carry meaningful implications for social work practice and mental health services in Saudi Arabia. Practitioners are encouraged to integrate loneliness screening into psychosocial assessments and to design community-based interventions that foster meaningful social connection and a sense of belonging. Financial support alone may not be sufficient to enhance life satisfaction among young adults. Income effects may reflect perceived gaps between aspirations and actual resources rather than absolute deprivation, suggesting that interventions should help youth understand available support resources and set realistic goals that align with their current circumstances. Future research should employ longitudinal designs to confirm causal pathways, examine potential mediating variables such as sense of belonging and social support, and expand the sample beyond the 18–25 age range to capture wellbeing trajectories across a broader span of adulthood.

## Data Availability

The raw data supporting the conclusions of this article will be made available by the authors, without undue reservation.
